# Entomopathogenic fungi in Portuguese vineyards soils: suggesting a ‘*Galleria*-*Tenebrio*-bait method’ as bait-insects *Galleria* and *Tenebrio* significantly underestimate the respective recoveries of *Metarhizium (robertsii)* and *Beauveria (bassiana)*

**DOI:** 10.3897/mycokeys.38.26790

**Published:** 2018-08-06

**Authors:** Lav Sharma, Irene Oliveira, Laura Torres, Guilhermina Marques

**Affiliations:** 1 CITAB – Centre for the Research and Technology of Agro-Environmental and Biological Sciences, University of Trás-os-Montes and Alto Douro, UTAD, Quinta de Prados, 5001-801, Vila Real, Portugal University of Trás-os-Montes and Alto Douro Vila Real Portugal; 2 CEMAT-IST-UL – Centre for Computational and Stochastic Mathematics, University of Lisbon, 1049-001 Lisbon, Portugal University of Lisbon Lisbon Portugal

**Keywords:** Biocontrol fungi, Functional diversity, Host-pathogen interaction, Hypocreales, Soil ecology, Vineyards

## Abstract

Entomopathogenic fungi (EPF) are the natural enemies of insect-pests. However, EPF recoveries can be influenced by the soil habitat-type(s) incorporated and/or the bait-insect(s) used. *Galleriamellonella* (GM) as bait-insect, i.e. ‘*Galleria*-bait’, is arguably the most common methodology, which is sometimes used solely, to isolate EPF from soils. Insect baiting using *Tenebriomolitor* (TM) has also been employed occasionally. Here 183 soils were used to estimate the functional diversity of EPF in Portuguese Douro vineyards (cultivated habitat) and adjacent hedgerows (semi-natural habitat), using the TM bait method. Moreover, to study the effect of insect baiting on EPF recovery, 81 of these 183 soil samples were also tested for EPF occurrences using the GM bait method. Twelve species were found in 44.26% ± 3.67% of the total of 183 soils. Clonostachysroseaf.rosea was found in maximum soils (30.05% ± 3.38%), followed by *Beauveriabassiana* (12.57% ± 2.37%), *Purpureocilliumlilacinum* (9.29% ± 2.14%) and *Metarhiziumrobertsii* (6.01% ± 1.75%). *Beauveriapseudobassiana* (*P* < 0.001), C.roseaf.rosea (*P* = 0.006) and *Cordycepscicadae* (*P*=0.023) were isolated significantly more from hedgerows, highlighting their sensitivities towards agricultural disturbances. *Beauveriabassiana* (*P* = 0.038) and *M.robertsii* (*P* = 0.003) were isolated significantly more using GM and TM, respectively. Principal component analysis revealed that *M.robertsii* was associated both with TM baiting and cultivated habitats, however, *B.bassiana* was slightly linked with GM baiting only. Ecological profiles of *B.bassiana* and *P.lilacinum* were quite similar while *M.robertsii* and C.roseaf.rosea were relatively distant and distinct. To us, this is the first report on (a) *C.cicadae* isolation from Mediterranean soils, (b) *Purpureocilliumlavendulum* as an EPF worldwide; and (c) significant recoveries of *M.robertsii* using TM over GM. Overall, a ‘*Galleria*-*Tenebrio*-bait method’ is advocated to study the functional diversity of EPF in agroecosystems.

## Introduction

Grape production and winemaking contribute significantly in many economies worldwide. However, vineyards attract many primary, secondary or tertiary insect pests (Gonçalves et al. 2017, Sharma et al. 2018). For example, one of the key insect-pest in vineyards is the European Grapevine Moth, *Lobesiabotrana* (Denis and Schiffermüller) (Lepidoptera: Tortricidae). It exhibits polyphagy and is distributed across Asia, Central Europe and the Mediterranean basin, USA, Chile and Argentina. It can reduce the total crop yield by 50% at the time of harvest in countries such as Portugal (Carlos et al. 2013). Finding strategies to control vineyards’ pests is of utmost importance especially from an economic point of view (Sharma et al. 2018).

With increased awareness towards the environment, biological methods to control crop pests such as biopesticides based on entomopathogenic fungi (EPF) have been receiving greater attention as alternatives to chemicals pesticides (Jaronski 2010). Many fungal species belonging to Hypocreales (Ascomycota) have shown insect pathogenicity and dwell in the soil for a significant part of their life cycle, outside the host. Protection from UV radiation and numerous adverse biotic and abiotic influences have made soil an excellent environmental reservoir for EPF (Keller and Zimmermann 1989). Therefore, studying soils for EPF diversity has been a common practice (Meyling and Eilenberg 2006, Quesada-Moraga et al. 2007, Goble et al. 2010, Rudeen et al. 2013, Muñiz-Reyes et al. 2014, Clifton et al. 2015, 2018).

Interestingly, the distribution of EPF in crop cultivated and semi-natural habitats, such as hedgerows, is always arguable. While some studies showed a higher abundance of *Beauveriabassiana* (Balsamo) Vuillemin in soils from hedgerows and *Metarhiziumanisopliae* (Metschnikoff) Sorokin in soils from cultivated fields (Meyling and Eilenberg 2006), others reported a higher abundance of *M.anisopliae* in marginal soils (Clifton et al. 2015). Habitat-specific preferences have also been noticed in the case of some EPF (Bidochka et al. 1998, Quesada-Moraga et al. 2007, Medo and Cagáň 2011, Medo et al. 2016). Knowing the differences in EPF abundances within different habitat-types is important in understanding which fungal species is suitable to and would proliferate in a particular habitat-type (Quesada-Moraga et al. 2007).

Insect baiting by *Galleriamellonella* Linnaeus (Lepidoptera: Pyralidae) or the ‘*Galleria*-bait method’ (Zimmermann 1986), is a renowned methodology for the isolation of EPF. The main advantage of the insect baiting method is that only entomopathogens are obtained selectively amongst other soil microbes (Vega et al. 2012). Studies in the past find insect baiting as an effective methodology for EPF isolation over culturing soil suspensions on selective media (Keller et al. 2003, Enkerli et al. 2004, Imoulan et al. 2011, Keyser et al. 2015). A selective medium can only be viewed as a semi-quantitative method for EPF isolation as they may provide a false picture of fungal diversity and density, leading to a biased view of many microbial systems (Scheepmaker and Butt 2010). The approach of using bait-insects *G.mellonella* along-with *T.molitor* for EPF isolations, instead of a selective media, has been previously employed (Vänninen 1996, Oddsdottir et al. 2010, Meyling et al. 2012).

Using different bait-insects sometimes may result in an occasional occurrence of a different, not so common EPF (Goble et al. 2010), however, to isolate the known EPF from soils, such as *Beauveria* and *Metarhizium*, the bait-insect *G.mellonella* has been the first choice as a bait-insect for the last three decades (Zimmermann 1986). Numerous investigations have relied only on this method of EPF isolation (Chandler et al. 1997, Bidochka et al. 1998, Ali-Shtayeh et al. 2003, Meyling and Eilenberg 2006, Quesada-Moraga et al. 2007, Sun and Liu 2008, Sun et al. 2008, Sevim et al. 2009, Fisher et al. 2011, Muñiz-Reyes et al. 2014, Pérez-González et al. 2014, Fernández-Salas et al. 2017, Gan and Wickings 2017, Kirubakaran et al. 2018). The bait-insect *Tenebriomolitor* Linnaeus (Coleoptera: Tenebrionidae) has also been used solely in some studies (Sánchez-Peña et al. 2011, Aguilera Sammaritano et al. 2016).

Fewer studies used these two bait-insects in parts or throughout their investigations (Hughes et al. 2004, Oddsdottir et al. 2010, Meyling et al. 2012). Hughes et al. (2004) noticed increased isolations of *Beauveria* and *Metarhizium* when bait-insects *G.mellonella* and *T.molitor*, respectively were used. This raised a question whether *Beauveria* and *Metarhizium* have preferences for the two common bait-insects *G.mellonella* and *T.molitor*? The main objectives of the above-mentioned and noteworthy studies were different. Hence, the observations of any insect species-specific differences remained obscure especially as no significant differences were observed.

Due to the lack of any study which focuses primarily on the differences of *Beauveria* and *Metarhizium* occurrences from soils while using *G.mellonella* and *T.molitor* bait-insects, some of the most recent and noteworthy studies, even those reported in the last few months, still use the *Galleria*-bait method as the standard (only) methodology to recover EPF from soils (Fernández-Salas et al. 2017, Gan and Wickings 2017, Kirubakaran et al. 2018). Keyser et al. (2015) compared the use of *T.molitor* against culturing soil samples over selective medium and a found a drastic contrast where the former was found highly effective over the latter. Although *T.molitor* has been used previously, still some very recent and interesting studies have, however, used *G.mellonella* and neglected the use of *T.molitor* even when the main objective was to understand the ecology of *Metarhizium* (Hernández-Domínguez and Guzmán-Franco 2017).

The influence of the use of *T.molitor* as a bait-insect to isolate EPF such as *Beauveria* and *Metarhizium*, if any, when compared with *G.mellonella*, remains an important question, especially after the observations of Hughes et al. (2004), as described earlier. Moreover, as different fungal entomopathogens are susceptible to different bait-insects as well as habitat-types, another important question, that might be of interest, is to understand what is the major factor(s), if any, that governs the recovery of common EPF such as *Beauveria* and *Metarhizium*.

Although there are previous reports on the EPF from different agroecosystems, the information on the functional diversity of EPF in vineyards is, however, very limited. The landscape of the Douro Wine Region (DWR) provides a good opportunity to understand the differences in EPF abundance and diversity amongst vineyards and adjacent hedgerows. Hence, the objectives of the work were to elucidate the effects of (1) habitat-types, i.e. cultivated soils of vineyards and semi-natural soils of nearby hedgerows and (2) bait-insects, i.e. *T.molitor* and *G.mellonella* on EPF while exploring (a) their recoveries, (b) ecological proximities and (c) the principal factors governing their presence in the soils of the vineyards of the DWR of Portugal. The focus of the investigation was to understand the functional fungal entomopathogenicity of soils.

## Methods

### Soil sampling

**Figure 1.**
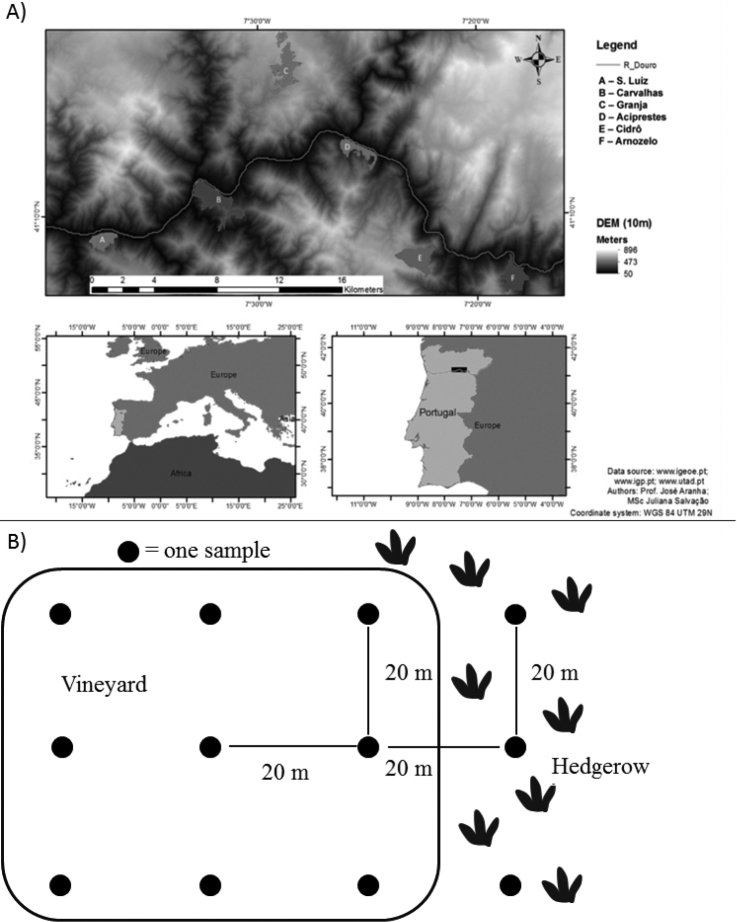
Geographic coordinates and altitudes of the farms and details of the soil sampling strategy adopted. **a** Details of the six farms of the Douro Wine Region, Portugal, which were considered in this study **b** Details of the soil sampling strategy from vineyards and adjacent hedgerows.

Soil samples were collected from six different farms of Portuguese DWR in September and October 2012, i.e. Arnozelo, Aciprestes, Carvalhas, Cidrô, Granja and S. Luiz. Details of geographic coordinates and altitudes of these farms are given in Fig. 1A. The sampling strategy was adapted from Klingen et al. (2002) and Goble (2010) and presented in Fig. 1B and the authors find it quite similar to that undertaken by Clifton et al. (2015). In brief, at each site, the surface litter was removed and the soil was dug to a depth of 20 cm with a soil core borer (width = 20 mm) at five places within 0.25 m^2^ area. All five sub-samples from one site were put in the same polyethylene bag and sealed with a rubber band. This mix of five subsamples was considered as one soil sample from a site. The next sampling site was at 20 m away and the soil borer was washed with 5% sodium hypochlorite (NaOCl) between the sites. In total, 183 soil samples were collected, out of which 155 were from vineyards and 28 were from adjacent hedgerows. Hedgerows were mainly composed of oaks (*Quercus* spp. L., Fagaceae) and pine (*Pinus* spp. L., Pinaceae) trees. Soil samples were brought inside the laboratory and were spread on a tray and left overnight for the moisture to be equilibrated with the room temperature. This was done to avoid infestation with entomopathogenic nematodes (EPN), if any, as suggested by Quesada-Moraga et al. (2007). Soil samples were always processed within 24 hours of spreading on to the trays. The number of soil samples collected from each farm is provided in Table 1.

**Table 1.** Occurrence frequency (% of positive samples) of entomopathogenic fungi Douro vineyards’ soils and adjacent hedgerows.

Species	Species occurrence in the whole farm (*F*wf)						%*F*v	%*F*h	%*F*overall	Previous reports
	S. Luiz	Carvalhas	Granja	Arnozelo	Aciprestes	Cidrô				
	(*N* = 51)	(*N* = 44)	(*N* = 26)	(*N* = 20)	(*N* = 20)	(*N* = 22)				
All species*	37.25	59.09	61.54	45	30	22.73	39.35	71.43	44.26	
* Beauveria bassiana *	15.69	11.36	15.38	10	15	4.55	12.26	14.29	12.57	Several
* Beauveria pseudobassiana *	1.96	6.82	–	10	–	–	–	21.43	3.28	Several
* Beauveria varroae *	–	–	–	5	–	–	–	3.57	0.55	Several
Clonostachys rosea f. rosea	19.61	45.45	42.31	25	20	22.73	25.81	53.57	30.05	Several
*Cordyceps* sp.	3.92	2.27	–	–	–	–	1.94	–	1.64	Several
* Cordyceps cicadae *	3.92	–	–	–	–	–	–	7.14	1.1	Several
* Lecanicillium aphanocladii *	3.92	–	–	–	–	–	1.29	–	1.1	Several
* Lecanicillium dimorphum *	3.92	2.27	–	–	–	–	1.94	–	1.64	Several
* Metarhizium robertsii *	3.92	2.27	30.77	–	–	–	7.1	–	6.01	Several
* Metarhizium guizhouense *	1.96	–	3.85	–	–	–	1.29	–	1.1	Several
* Purpureocillium lavendulum *	–	2.27	–	–	–	–	0.65	–	0.55	This study
* Purpureocillium lilacinum *	9.8	13.64	15.38	10	–	–	10.32	3.57	9.29	Several

*, 12 different fungal species in total.

*N*: Number of soil samples.

%*F*v: Percentage frequency of the number of soil samples harbouring a particular fungal species isolated from 155 soil samples from vineyards’ soils of six farms.

%*F*h: Percentage frequency of the number of soil samples harbouring a particular fungal species isolated from 28 soil samples from hedgerows’ soils of six farms.

%*F*overall: Percentage frequency of the number of soil samples harbouring a particular fungal species isolated from all 183 soil samples from six farms.

*F*wf: Percentage frequency of the number of soil samples harbouring a particular fungal species isolated from total number of soil samples collected from that respective farm.

### Insect baiting

Two hundred and fifty grams (g) of sieved soil was put in a plastic bowl with small holes on the cap for ventilation. A total of 183 soil samples were used to compare the effect of habitat-type on fungal isolations. For each soil sampling site, four such bowls, i.e. 1 kg of the soil was analysed in total and four late instar *T.molitor* larvae were put in each of these bowls, i.e. the total number of larvae used (*n*) = 16. To study the effect of insect baiting, 81 of the total 183 soil samples were baited with late instar larvae of *G.mellonella* (*n* = 8) and *T.molitor* (*n* = 8) similarly, such that the total number of larvae, irrespective of the bait-insect type, remained same, i.e. *n* = 16. These 81 soil samples were from the three farms with a relatively diverse landscape, i.e. S. Luis, Carvalhas and Granja, as reported by Carlos et al. (2013). Hence, these farms were chosen to enhance the fungal diversity, in theory. This would facilitate studying the effect of insect baiting on a rather diverse group of EPF. *Galleriamellonella* was given heat shock by immersing in 56 °C water prior to baiting, to reduce the tendency of silk web formation within soil samples as suggested by Meyling and Eilenberg (2006). Bowls were kept in an environmental chamber (Panasonic MLR-352H-PE) at a temperature of 22 °C and relative humidity of 85%, in the dark. Bowls were frequently inverted, shaken gently and kept upside down for the total incubation period of three weeks as per Meyling and Eilenberg (2006).

### Fungal isolation and screening

The presence of insect cadavers was observed every day for the first week and every second day for the remaining two weeks. Everyday monitoring was necessary for the first week as death by EPN, if any, generally was caused within the first three days of larvae incubation in soils, although slightly delayed infection cannot be neglected. The schedules were monitored rigorously and the insect cadavers were observed quite carefully. Any cadavers with a foul smell were constantly discarded. Obtained cadavers were washed with 1% NaOCl for three minutes, followed by three distinct washes of 100 ml sterilised distilled water for three minutes each. It was done to isolate only the fungi which have penetrated the insect cuticles and proliferated within the insect haemocoel or have been ingested into the haemocoel. The cadavers were subsequently cultured on to potato dextrose agar (PDA) (Liofilchem) plates supplemented with 0.1 g/l streptomycin (Acros) and 0.05 g/l tetracycline (Acros). In cases of mixed infections or inhibited fungal growth, cadavers were cultured on to oatmeal agar (OA) supplemented with 0.5 g/l chloramphenicol (Acros) and 0.6 g/l cetyl trimethyl ammonium bromide (CTAB) (Sigma) as described in Posadas et al. (2012). Repeated culturing on OA or/and Sabouraud dextrose agar (SDA) (Prolabo) was undertaken until the pure culture of fungus was obtained. Plates were repeatedly observed through a low magnifying stereomicroscope (Olympus SZX9, 40X magnification) and, if any emergence of nematodes were observed, they were discarded no matter if a fungal growth was present or absent. Any possibility of cross-contamination or external contamination was carefully monitored as described by Steinwender et al. (2014). No colony forming units (CFUs) were observed in any of the tests for contaminations. To confirm Koch’s postulates, all the obtained fungi were tested using bioassays for pathogenicity against the larvae from which they were isolated. The method was initially described by Ali-Shtayeh et al. (2003), however, a modified protocol was used as described in Sun and Liu (2008) and Goble et al. (2010). The fungi found pathogenic to insect larvae were considered further in this study.

### Fungal identification and DNA extraction

The appearance on the infected larvae and morphological characteristics were used as the preliminary identification of fungi. Morphological characteristics that were used for identification are described in a taxonomic key (Domsch et al. 2007). For molecular identification, DNA was extracted from fungal mycelium as described earlier by Möller et al. (1992). Moreover, the protocol was optimised for hard-to-crush mycelium and spores as in Sharma et al (2018). The fungal internal transcribed spacer (ITS) region was amplified using the forward primer ITS1-F (5’-CTTGGTCATTTAGAGGAAGTAA-3’) and reverse primer ITS4 (5’-TCCTCCGCTTATTGATATGC-3’) (Gardes and Bruns 1993). The PCR reaction was performed as described in Yurkov et al. (2015). Primers used for PCR reactions were also used for amplicon sequencing. Sequences were edited using BioEdit 7.2.1 (Hall 1999) and further aligned using MAFFT version 7 (Katoh and Standley 2013) to validate polymorphisms amongst sequences. Obtained ITS sequences from EPF were aligned with those from the respective type strain sequences using BLASTn and the identity results are shown in Suppl. material 1: Table S4. Newly generated sequences were submitted to EMBL nucleotide sequence database and the accession numbers are provided in the Suppl. material 1: Table S4.

### Data analyses

Fungal species richness (*S*) was compared in terms of habitat-types and bait-insects used for isolation. Jaccard’s similarity coefficients (*J*) for fungal species shared between different habitats and bait-insects were measured as described in Garrido-Jurado et al. (2015). *J = a/(a+b+c)*, where “*a*” represents the number of species occurring in both variables, “*b*” represents the number of species occurring only in variable 1 and “*c*” represents the number of species occurring only in variable 2. *J* can range between 0 (no shared species) to 1 (all shared species). Software IBM SPSS Statistics 22 was used to perform statistical data processing. Infections were counted qualitatively per site, i.e. whether a particular fungus infected one or several insect larvae of the same bait-insect, it was registered as one infection for that fungal species, as described in Klingen et al. (2002) and Goble et al. (2010). Therefore, effects of soil habitat-types and bait-insects are counted in accordance with the number of soil samples found harbouring a fungal species as in Klingen et al. (2002), Goble et al. (2010) and Clifton et al. (2015). Data were treated using Fisher’s exact test as it gives the exact *P* value for a 2×2 contingency table (https://www.graphpad.com/). Besides, farm type variations could only be analysed using the χ^2^ (chi-square) test and Monte Carlo simulations were used in case the cells have the expected count of less than 5. Data used for different analyses, i.e. (1) effect of bait-insect type on the occurrence of EPF; (2) effect of habitat-type (hedgerows vs. vineyards) on EPF occurrence; and (3) effect of farm type on EPF occurrence, are provided in detail within the Suppl. material 1: Tables S1, S2 and S3, respectively. To compare possible factors which may influence fungal recoveries, a principal component analysis (PCA) was performed. The PCA was conducted on the mean-centred and scaled data in order to investigate the discriminations of the obtained fungal species. For the PCA plots, only those soils samples were considered where both the bait-insects, i.e. *T.molitor* and *G.mellonella* were used, i.e. soils from the farms S. Luis, Carvalhas and Granja (Suppl. material 1: Table S1). Fungi with isolation frequencies of <10% from either vineyards or hedgerows were considered as rare EPF. Hierarchical clustering was then employed to investigate the degree of similarities of fungal isolations based on their ecological proximities, i.e. in terms of habitat-type and bait-insect type. The resulting dendrogram was obtained based on the Euclidean distance and Ward aggregation method as in Sharma et al. (2018). Software R 3.4.2 was used to generate PCA plots and hierarchical clustering.

## Results

### Overall fungal species abundance

The total numbers of soil samples used were 183 and the number of soil samples found positive (*N*) with any EPF were 81, i.e. 44.26% ± 3.67% soils. A total of 12 different species were observed (Table 1). Clonostachysroseaf.rosea (Link) Schroers, Samuels, Seifert & Gams was found in the maximum number of soil samples i.e. 30.05% ± 3.38% (*N* = 55), followed by *B.bassiana* (12.57% ± 2.37% (*N* = 23)), *Purpureocilliumlilacinum* (Thom) Luangsa-ard, Houbraken, Hywel-Jones & Samson (9.29% ± 2.14% (*N* = 17)) and *Metarhiziumrobertsii* Bischoff, Rehner & Humber (6.01% ± 1.75% (*N* = 11)).

Isolations of *Beauveriapseudobassiana* Rehner & Humber (3.38% ± 1.31% (*N* = 6)), *Cordyceps* sp. Fries (1.64% ± 0.94% (*N* = 3)), *Lecanicilliumdimorphum* (Chen) Zare & Gams (1.64% ± 0.94% (*N* = 3)), *Cordycepscicadae* (Miq.) Massee (1.10% ± 0.77% (*N* = 2)), *Lecanicilliumaphanocladii* Zare & Gams (1.10% ± 0.77% (*N* = 2)), *Metarhiziumguizhouense* Chen & Guo (1.10% ± 0.77% (*N* = 2)), *Beauveriavarroae* Rehner & Humber (0.55% ± 0.54% (*N* = 1)) and *Purpureocilliumlavendulum* Perdomo, García, Gené, Cano & Guarro (0.55% ± 0.54% (*N* = 1)) were also observed (Table 1). The fungal occurrence was the highest in the farm Granja, i.e. 61.54% ± 9.54% (*N* = 16), followed by Carvalhas (59.09% ± 7.4% (*N* = 26)), Arnozelo (45% ± 11.12% (*N* = 9)), S. Luiz (37.25% ± 6.77% (*N* = 19)), Aciprestes (30% ± 10.24% (*N* = 6)) and Cidrô (22.73% ± 8.93% (*N* = 6)) (Table 1).

### Effect of insect baiting on fungal isolation

**Figure 2.**
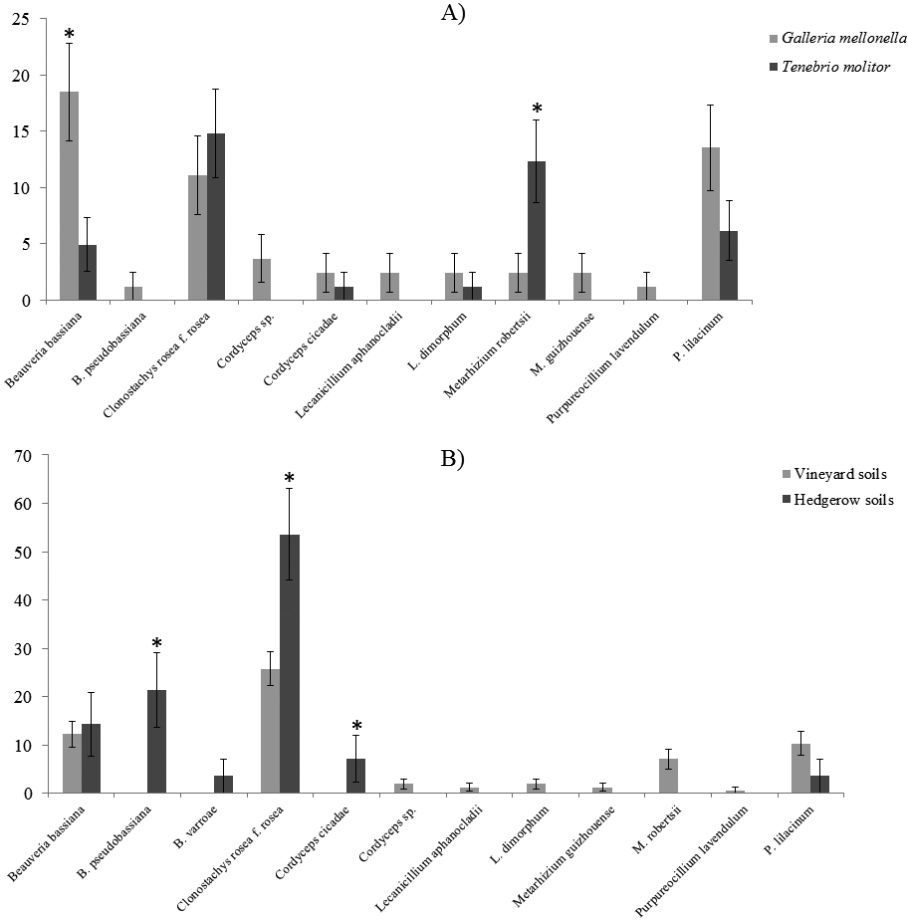
Effect of insect baiting and habitat-type on the isolation of the entomopathogenic fungi. **a** Occurrence (% of soil samples ± SE) of entomopathogenic fungi when different bait-insects were incorporated **b** Occurrence (% of soil samples ± SE) of entomopathogenic fungi when soils were collected from different habitat-types. Bars with asterisk (*) show significant isolations, i.e. (*P*<0.05).

To test the effect of insect baiting on EPF recoveries, bait-insects *G.mellonella* (*n* = 8) and *T.molitor* (*n* = 8) were employed on 81 soil samples from the three farms which had quite diverse landscapes, i.e. S Luiz, Carvalhas and Granja. Hence, in total, 16 larvae from two different bait-insects were used. Eleven EPF species were observed amongst the three farms and a few significant differences were detected within fungal recoveries (Fig. 2A, Suppl. material 1: Table S1). Significantly more soil samples were found positive for *B.bassiana* when *G.mellonella* was used as a bait-insect, i.e. 15 isolates (18.52% ± 4.31%) than *T.molitor*, i.e. 4 isolates (4.94% ± 2.4%) (*P* = 0.038). On the contrary, isolation of *M.robertsii* was increased significantly by *T.molitor*, i.e. 10 isolates (12.35% ± 3.65%) compared to *G.mellonella*, i.e. 2 isolates (2.47% ± 1.72%) (*P* = 0.003).

Clonostachysroseaf.rosea was isolated more often by *T.molitor*, i.e. (14.81% ± 3.94% (*N* = 12)) than by *G.mellonella*, i.e. (11.11% ± 3.49% (*N* = 9)). Moreover, *T.molitor* specific isolations were noticed for *M.guizhouense*, i.e. 2.47% ± 1.72% (*N* = 2). However, *G.mellonella* recovered more *C.cicadae* and *L.dimorphum*, i.e. 2.47% ± 1.72% (*N* = 2) than 1.23% ± 1.22% (*N* = 1) by *T.molitor*, in cases of both the fungi. *Galleriamellonella* specific isolations for *Cordyceps* sp. (3.79% ± 2.09% (*N* = 3)), *L.dimorphum* (2.47% ± 1.72% (*N* = 2)) and *P.lavendulum* (1.23% ± 1.22% (*N* = 1)) were also recorded (Fig. 2A, Suppl. material 1: Table S1). Overall, using *G.mellonella* yielded slightly more fungal species (i.e. *S* = 10) than *T.molitor* (i.e. *S* = 7) (Table 2).

**Table 2.** Entomopathogenic fungal species richness and similarities amongst isolations from different habitat-types and bait-insects.

	Observed species (*S*, richness)		Jaccard coefficient (*J*)
	Vineyards	Hedgerows	*J* (habitat)
Soil(GM)	8	5	0.435
Soil(TM)	6	4	0.41
Soil*	9	6	0.44
	* Galleria mellonella *	* Tenebrio molitor *	*J* (bait-insect)
Soil(V)	8	6	0.39
Soil(H)	5	4	0.35
Soil^#^	10	7	0.39

Soil(GM): soil samples baited by *Galleriamellonella* larvae; Soil(TM): soil samples baited with *Tenebriomolitor* larvae; Soil(V): soil samples collected from vineyards; Soil(H): soil samples collected from vineyards.

*, overall samples irrespective of bait-insect type.

^#^, overall samples irrespective of habitat-type.

Note: Jaccard coefficient for similarity amongst habitat types, *J* (habitat) = a/(a + b + c), where ‘‘a’’ is the number of species occurring in both habitats, ‘‘b’’ is the number of species specific to vineyards and ‘‘c’’ is the number of species specific to hedgerows. *J* ranges from 0 (no shared species amongst habitats) to 1 (all species are shared amongst habitats). Similar calculations were done for *J* (bait-insect), where values corresponded to observed fungal species when different bait-insects were used.

### Effect of habitat-types on fungal isolation

To study the habitat type variation, 183 soil samples from all the six farms were considered, i.e. 155 from vineyards and 28 from hedgerows. As two different bait-insects, *G.mellonella* and *T.molitor*, were used in the three farms, i.e. S. Luiz, Carvalhas and Granja and only one bait-insect *T.molitor* was used in the other farms, i.e. Aciprestes, Arnozelo and Cidrô, the numbers of bait-insects larvae used to study the habitat-type variations in each farm were kept constant, i.e. *n* = 16.

Out of 155 soil samples from vineyards, a total of nine EPF species were observed in 61 vineyards’ soils, i.e. 39.35% ± 3.81% soils were found harbouring at least one EPF. Six fungal species were observed solely from vineyards, i.e. *Cordyceps* sp. (1.94% ± 1.1% (*N* = 3)), *L.aphanocladii* (1.29% ± 0.9% (*N* = 2)), *L.dimorphum* (1.94% ± 1.1% (*N* = 3)), *M.robertsii* (7.10% ± 2.06% (*N* = 11)), *M.guizhouense* (1.29% ± 0.9% (*N* = 2)) and *P.lavendulum* (0.65% ± 0.64% (*N* = 1)). Although *M.robertsii* was isolated only from vineyards, however, recoveries were not significant (*P* = 0.220). Three species, i.e. *P.lilacinum*, C.roseaf.rosea and *B.bassiana* were shared amongst both habitat-types. *Purpureocilliumlilacinum* was isolated more frequently from vineyard soils i.e. 16 isolates (10.32% ± 2.44%) than hedgerows, i.e. 1 isolate (3.57% ± 3.50%), however, non-significantly (*P* = 0.228) (Fig. 2B, Table 1).

*Beauveriabassiana* was slightly more abundant in hedgerows, i.e. 4 isolates in 28 samples (14.29% ± 6.61%) than in vineyards, i.e. 19 isolates in 155 samples (12.26% ± 2.63%), although differences were not significant (*P* = 0.759) (Table 1), (Fig. 2B). Clonostachysroseaf.rosea was also more frequent in hedgerows, i.e. in 15 of the 28 samples (53.57% ± 9.42%) than in vineyards i.e. 40 of the 155 samples (25.81% ± 3.51%) (*P* = 0.006). Moreover, *B.pseudobassiana* only occurred in hedgerows, i.e. 6 isolates (21.43% ± 7.75%) (*P*<0.001). *Beauveriavarroae* (3.57% ± 3.50% (*N* = 1)) and *C.cicadae* (7.14% ± 4.86% (*N* = 2)) (*P* = 0.023) were also noticed in hedgerows’ soils only (Fig. 2B). Overall, significantly higher number of soil samples were found positive for EPF in hedgerows, i.e. 20 isolates in 28 samples (71.43% ± 8.53%), than in vineyards, i.e. 61 isolates in 155 samples (39.35% ± 3.92%) (*P*<0.001) (Table 1). However, fungal species richness (*S*) was higher in soils from vineyards, i.e. *S* = 9 than from hedgerows, i.e. *S* = 6 (Table 2). Additional information on the habitat-types variations is shown in Suppl. material 1: Table S2.

### Farm type variation

Those EPF which were recovered from all six farms using *T.molitor* larvae (*n* = 16) only, were considered to study the farm type variations. This was done to avoid any bias as *T.molitor* was the bait-insect used in all six farms. Nine EPF species were recovered and C.roseaf.rosea was isolated significantly more from Carvalhas, i.e. from 18 of the total of 48 soil samples collected from the respective farm (*N* = 18/48), (37.5% ± 6.98%) (χ^2^ = 12.981, df = 5, *P* = 0.0024). *Metarhiziumrobertsii* was isolated more frequently from Granja (*N* = 8/11) (72.72% ± 13.4%) (χ^2^ = 33.657, df = 5, *P*<0.001). *Beauveriabassiana* was found distributed throughout all farms, i.e. Aciprestes (*N* = 3/20) (15% ± 7.98%); Arnozelo (*N* = 2/20) (10% ± 6.7%), S. Luiz (*N* = 3/51) (5.88% ± 3.29%), Carvalhas (*N* = 2/44) (4.55% ± 3.14%), Cidrô (*N* = 1/22) (4.55% ± 4.44%) and Granja (*N* = 1/26) (3.85% ± 3.77%). *Purpureocilliumlilacinum* was found in four of the six farms, i.e. Arnozelo (*N* = 2/20) (10% ± 6.7%), Carvalhas (*N* = 2/44) (4.55% ± 3.14%), S. Luiz (*N* = 2/51) (3.92% ± 2.71%) and Granja (*N* = 1/26) (3.85% ± 3.77%). More details about other fungi are in the supplementary information (Suppl. material 1: Table S3).

### Ecological proximities based dendrogram and principal recovery factors

**Figure 3.**
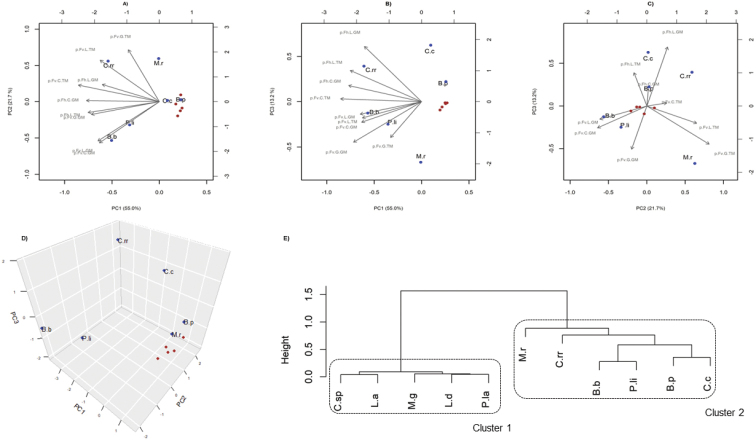
Principal component analysis (PCA) and hierarchical clustering of the observations based on the fungal isolations. **a**PC1 vs. PC2. **b**PC1 vs. PC3. **c**PC2 vs. PC3. **d** PCA 3D plot **e** Hierarchical clustering dendrogram to access the ecological proximities of obtained fungi based on their respective isolation profiles. Software R 4.3.2 was used to obtain the PCA plots and the hierarchical clustering. There was no fungal isolation from hedgerows from the farm Granja when bait-insect *T.molitor* was used and hence, it could not be included in any of the analysis which relies on proportions, i.e. PCA plots, hierarchical clustering. To reduce any bias, the authors also discarded the soil samples (*N*=1) which yielded the fungal isolations, when *G.mellonella* was used, from the hedgerows of the farm Granja. The blue balls represent relatively more frequent EPF, i.e. *Beauveriabassiana*, *Beauveriapseudobassiana*, Clonostachysroseaf.rosea, *Cordycepscicadae*, *Purpureocilliumlilacinum* and *Metarhiziumrobertsii*. The red balls represent other fungi such as *Cordyceps* sp., *Lecanicilliumaphanocladii*, *Lecanicilliumdimorphum*, *Metarhiziumguizhouense* and *Purpureocilliumlavendulum*. Hierarchical clustering based dendrogram classified isolated EPF into two clusters, i.e. rarely occurring EPF (cluster 1) and relatively more frequent EPF (cluster 2). Abbreviations used are: *Beauveriabassiana* (B.b), *Beauveriapseudobassiana* (B.p), *Cordycepscicadae* (C.c), *Cordyceps* sp. (C.sp), *Lecanicilliumaphanocladii* (L.a), *Lecanicilliumdimorphum* (L.d), *Metarhiziumguizhouense* (M.g), *Purpureocilliumlavendulum* (P.la), *Purpureocilliumlilacinum* (P.l), Clonostachysroseaf.rosea (C.rr) and *Metarhiziumrobertsii* (M.r).

A PCA was performed on the EPF recovery data from the 81 soils of the three farms, i.e. S. Luis, Carvalhas and Granja, where both habitat-types and bait-insects were incorporated. This kind of analysis was done to understand which element(s), i.e. bait-insect(s) and/or habitat-type(s), governs the recovery of the EPF. Using PCA, 89.9% of the variance among fungal recoveries could be described by the three principal components, i.e. PC1 (55%), PC2 (21.7%) and PC3 (13.2%) (Fig. 3A, B, C). Second principal component (PC2) was slightly dominated by the type of bait-insect used (Fig. 3A, C). The occurrences of *B.bassiana* and *P.lilacinum* were slightly and marginally governed by insect baiting using *G.mellonella*, respectively. However, the isolations of C.roseaf.rosea and *M.robertsii* were slightly and mainly governed by baiting using *T.molitor*, respectively (Fig. 3A–D). Third principal component (PC3) could distinctly separate the two habitat-types (Fig. 3B, C). The isolations of C.roseaf.rosea were mostly governed by semi-natural habitats. However, *M.robertsii* and *P.lilacinum* were highly and slightly influenced also by cultivated habitats, respectively. *Codycepscicadae* recovery was governed only by hedgerows (Fig. 3A–D). Hierarchical clustering dendrogram of the ecological proximities of fungi, after profiling their recoveries from bait-insects and habitat-types, placed *B.bassiana* and *P.lilacinum* closer, while C.roseaf.rosea and *M.robertsii* were quite different and distinct (Fig. 3E). Moreover, the dendrogram also separated rare EPF, i.e. those with an isolation frequency of <10% from either of the habitat-types (cluster 1), from relatively more frequent EPF (cluster 2) (Fig. 3E).

## Discussion

### Insects baiting of soils for EPF recovery

Considering the number of soil samples and the objectives, this study was comparable with others on EPF occurrence and diversity (Tarasco et al. 1997, Klingen et al. 2002, Ali-Shtayeh et al. 2003, Quesada-Moraga et al. 2007, Sun et al. 2008, Imoulan et al. 2011, Schneider et al. 2012). The ‘*Galleria*-bait method’, i.e. using *G.mellonella* for EPF recovery from soils, was described by Zimmermann in the year 1986 (Zimmermann 1986). Since then it has been used quite often in numerous studies as the only method for EPF isolations, in the past three decades (Chandler et al. 1997, Bidochka et al. 1998, Ali-Shtayeh et al. 2003, Meyling and Eilenberg 2006, Quesada-Moraga et al. 2007, Sun and Liu 2008, Sun et al. 2008, Sevim et al. 2009, Fisher et al. 2011, Muñiz-Reyes et al. 2014, Pérez-González et al. 2014, Fernández-Salas et al. 2017, Gan and Wickings 2017, Kirubakaran et al. 2018). Similarly, in few other studies, insect baiting using *T.molitor* is the only method used for the EPF recovery (Sánchez-Peña et al. 2011, Steinwender et al. 2014).

### Fungal recovery using *Galleriamellonella* bait-insect

*Beauveriabassiana* was isolated significantly more from *G.mellonella* (*P* = 0.038) (Fig. 2A) as in South Africa by Goble et al. (2010). Klingen et al. (2002) found insect-specific isolations of *B.bassiana* by *G.mellonella* in Norway. Studies in Iceland and Greenland also concluded that *B.bassiana* was isolated more often by *G.mellonella* (Oddsdottir et al. 2010, Meyling et al. 2012). Many previous reports are available on the recovery of different fungi from *G.mellonella*, for example, *C.cicadae* (Barker and Barker 1998), *P.lilacinum* (Imoulan et al. 2011), *Lecanicillium* spp. (Hypocreales: Cordycipitaceae) (Asensio et al. 2003, Meyling and Eilenberg 2006), as in the present study. To our knowledge, this study reports the first isolation of *P.lavendulum* from an insect.

### Fungal recovery using *Tenebriomolitor* bait-insect

In the present study, insect-specific isolation of *M.guizhouense* and significant isolation of *M.robertsii* was reported from *T.molitor* (*P* = 0.003) (Fig. 2A) (Suppl. material 1: Table S1). Comparing *G.mellonella* and *T.molitor*, insect-specific isolation of *Metarhizium* has been reported using the latter (Oddsdottir et al. 2010). Hughes et al. (2004) found that, out of the 20 soils sampled, 15 harboured *Metarhizium* when *T.molitor* was used as bait-insect, compared with just four when *G.mellonella* was used. *Metarhizium* was found to be the most abundant EPF in the soils from the tropical forests of Panama, although the soils were collected within 5 m from the nest of leaf-cutting ants (insect host) which possibly increased EPF recovery. Nonetheless, the major drawback of the study was that a very limited number of soil samples were used and the results were not analysed statistically (Hughes et al. 2004). In the present study, 81 soil samples were used to study the effect of insect baiting on EPF recovery. Moreover, a random selection of soil samples was promoted to reduce any bias for an enhanced EPF recovery and to maintain a practical scenario where no prior information on the presence of insect-host is necessary.

To our knowledge, this is the first report on the significantly higher recovery of *M.robertsii* by *T.molitor* when compared with that from *G.mellonella*. *Galleria*-bait is still a widely used method to isolate EPF from soils. Even the most recent reports, i.e. those reported in the past few months, overlook the use of *T.molitor* while studying with ecologies of EPF such as *Metarhizium* (Fernández-Salas et al. 2017, Gan and Wickings 2017, Hernández-Domínguez and Guzmán-Franco 2017, Kirubakaran et al. 2018). This study signifies that the use of both of the bait-insects is more important than considered before and *T.molitor* should always be used along with *G.mellonella*, especially when *Metarhizium* is being isolated from soils. Enhanced recovery of *Metarhizium* from *T.molitor* could be due to the higher sensitivity of the insect towards this fungus. Vänninen et al. (2000) found that even after three years post application, *M.anisopliae* could kill over 80% of the *T.molitor* baited in soils from different places.

### Entomopathogenic fungal communities within hedgerows’ soils (semi-natural habitat)

In this study, 15.3% of the total soil samples were from hedgerows, which were comparable with 20.5% of the soil samples from hedgerows examined by Meyling and Eilenberg (2006). *Beauveriabassiana* was slightly more abundant in hedgerows than in vineyards (Table 1), (Fig. 2B). Some previous studies also did not report any significant habitat preference for *B.bassiana* (Klingen et al. 2002, Quesada-Moraga et al. 2007). Only the soils from hedgerows could lead to the isolation of *B.pseudobassiana* and it was significant (*P*<0.001) (Fig. 2B). This finding agreed with Meyling and Eilenberg (2007), who found *B.pseudobassiana* only in hedgerows. *Cordycepscicadae* was also isolated in significant amounts from hedgerows (*P* = 0.023) (Fig. 2B). Barker and Barker (1998) reported that *C.cicadae* isolations were restricted to forest soils (i.e. less disturbed soils). To our knowledge, this is the first report on the isolation of *C.cicadae* from Mediterranean soils. Clonostachysroseaf.rosea was isolated more from less disturbed (i.e. orchard) soils than intensively disturbed (i.e. field crops) soils in this study as in Sun et al. (2008).

A possible reason of higher occurrence of *B.bassiana* and the habitat-specific occurrence of *B.pseudobassiana* and *B.varroae* in hedgerows could be the relatively higher dependence of *Beauveria* on secondary infections on insect hosts, as hedgerows are expected to host rather diverse insect communities (Goble et al. 2010). Besides, factors such as reduced ultra-violet radiation and temperatures, increased humidity and long-term environmental stability could also lead to an increased viability of these fungal spores (Meyling et al. 2009). Mycoparasitism, a characteristic of *B.bassiana* (Vega et al. 2009) and *C.rosea* (Keyser et al. 2016), could provide dominance amongst opportunistic saprophytes in hedgerows.

### Entomopathogenic fungal communities in vineyards (cultivated habitat)

Although *Purpureocilliumlilacinum* and *M.robertsii* were isolated more from vineyards’ soils, the results were, however, non-significant, i.e. *P* = 0.228 and *P* = 0.220 (Fig. 2B). Moreover, two strains of *M.guizhouense* were also isolated only from vineyards (Table 1). *Purpureocilliumlilacinum* could tolerate a wide range of temperatures, from 8 °C to 38 °C and pH (Roumpos 2005). As these properties provide robustness against agricultural disturbances, according to Wei et al. (2009), *P.lilacinum* is the most widely tested fungus under field conditions. Higher isolations of *Metarhizium* spp. from crop cultivated lands in Spain and Mexico have been reported (Quesada-Moraga et al. 2007, Sánchez-Peña et al. 2011). Tillage seemed to distribute *Metarhizium*CFUs evenly throughout the field which subsequently increases chances of fungal recovery from different sites (Kepler et al. 2015).

Fungal species richness (*S*) was higher in soils from vineyards, i.e. *S* = 9 than hedgerows, i.e. *S* = 6 (Table 2). Few genera mentioned in Table 1 were previously reported to be isolated more often from relatively more disturbed soils, for example, *Lecanicillium* (Meyling and Eilenberg 2006). Moreover, Sun et al. (2008) found higher species richness in soils of crop fields than from orchards soils (i.e. less disturbed soils), as in the present study.

More diverse fungal species in cultivated soils is not surprising. Practices such as ploughing, reseeding and fertilising increase environmental patches and niche availability for EPF and subsequently increase fungal diversity (Sun et al. 2008). The higher organic matter also increases biological activity in the soil which positively affects the presence of saprophytic fungi which lead to lesser organic resources for EPF and therefore, reduced survivability (Goble et al. 2010).

### Factors, ecological proximities and hierarchical clustering dendrogram of fungi

Studies on the EPF ecology in soils consider either different bait-insects or habitat-types or both, as discussed earlier. Principal component analysis was done to understand the most important factor, if any, that governs the recoveries of EPF. It was found that isolations of *B.bassiana* were slightly governed by baiting with *G.mellonella*, irrespective of the habitat-type incorporated (Fig. 3A, C, D). However, the isolations of *M.robertsii* were influenced both by the cultivated habitat-type as well as by baiting with *T.molitor* (Fig. 3A–D). The ecological proximities of *B.bassiana* and *P.lilacinum* could be explained as *P.lilacinum* was isolated more frequently from vineyard soils than from hedgerows and *B.bassiana* isolations were almost equal from vineyards to those from hedgerows (Figs 2B, 3D, E). Moreover, the bait-insect *G.mellonella* favoured *P.lilacinum* and *B.bassiana* isolations (Fig. 2A). Distinct profiles of C.roseaf.rosea and *M.robertsii* suggest their unique ecologies in terms of habitat-type and bait-insect preferences (Fig. 3D, E). The main advantage of fungal profiling by hierarchical clustering based dendrogram is that those EPF which were not isolated in this study can also be investigated for their roles in the biological control of interest pests in agroecosystems, if they exhibit similar ecological profiles (Sharma et al. 2018).

### Fungal abundance and diversity

Entomopathogenic fungi was observed in 44.26% ± 3.67% of the soil samples and it was comparable to previous studies in Finland (38.6%) (Vänninen 1996), Palestine (33.6%) (Ali-Shtayeh et al. 2003), Alicante province, Spain (32.8%) (Asensio et al. 2003), South Africa (21.53%) (Goble et al. 2010), UK (17.6%) (Chandler et al. 1997) and southern Italy (14.9%) (Tarasco et al. 1997). More diverse fungal species were found in the present study when compared with the other studies in Mediterranean regions, for example, in Italy (Tarasco et al. 1997), Spain (Asensio et al. 2003, Quesada-Moraga et al. 2007, Garrido-Jurado et al. 2015), Turkey (Sevim et al. 2009) and Morocco (Imoulan et al. 2011). Different studies suggest that *Metarhizium* spp. are either absent (Ali-Shtayeh et al. 2003, Oliveira et al. 2012) or less prevalent in the Mediterranean region (Tarasco et al. 1997, Asensio et al. 2003, Quesada-Moraga et al. 2007, Garrido-Jurado et al. 2015). Surprisingly, Garrido-Jurado et al. (2015) reported just four isolates of *M.robertsii* from 270 soil samples in Spain which was quite a small number compared with the 11 isolates from 183 soil samples found in the present study. Occasional isolations of many species were noticed in the present study and, according to our knowledge, this is the first isolation of entomopathogenic strains of *B.varroae*, *L.aphanocladii*, *L.dimorphum*, *M.robertsii* and *M.guizhouense* in Portugal.

## Conclusion

Entomopathogenic fungi have been known for their potential as insect biocontrol agents and recent studies focus on their use for conservation biological control. However, the information about their ecology in vineyards is very limited. The main aim of the research was to analyse functional fungal entomopathogenicity of the soils of DWR in Portugal. It was found that different habitat-types and bait-insects have significant effects on the isolation of certain EPF species. Species richness and abundance differed amongst soil habitats. Clonostachysroseaf.rosea is a renowned mycoparasite and, recently, it has been tested positive for endophytism and entomopathogenicity. The higher recovery of C.roseaf.rosea from semi-natural habitats suggests its use in less disturbed soils. Moreover, hedgerow-specific isolation of *B.pseudobassiana* points to its inability to withstand harsher conditions in cultivated soils. The first isolation of *C.cicadae* as an EPF from Mediterranean soils supports its biocontrol potential in this climate, at least in less-disturbed habitats. Therefore, these properties should be capitalised accordingly. Principal component analysis could decipher that baiting, using *G.mellonella*, influence the isolations of *B.bassiana*, irrespective of the habitat-type incorporated. However, *M.robertsii* isolations were highly governed by the cultivated habitat-type as well as by the use of *T.molitor* as bait-insect. Overall, it was observed that DWR harbour various EPF which could be used as potential biocontrol agents for vineyard pests such as the European Grapevine Moth and understanding the functional ecology of EPF could help in using them more efficiently.

Although *T.molitor* has been used previously on a few occasions, still many of the recent studies, even those conducted in the past few months, overlook the use of *T.molitor* when dealing with EPF and especially *Metarhizium* ecology. While these studies bring a significant advancement to our knowledge in EPF ecology, they suffer from the lack of any concrete study which highlights the significant limitations of using the ‘*Galleria*-bait method’ alone to isolate *Metarhizium* from soils. As *G.mellonella* was a significantly better bait-insect for isolating *B.bassiana*, therefore, the combined use of *G.mellonella* and *T.molitor* is indispensable for a more complete understanding of EPF diversity and distribution within a region. In this study, the authors modify the existing ‘*Galleria*-bait method’ and propose the use of the ‘*Galleria*-*Tenebrio*-bait method’ for future studies in this area.

## Supplementary materials

Supplementary material 1 Supplementary tables Data type: species data This is an open access article distributed under the terms of the Creative Commons Attribution License (CC BY 4.0), which permits unrestricted use, distribution, and reproduction in any medium, provided the original author and source are credited.Lav Sharma, Irene Oliveira, Laura Torres, Guilhermina Marques
